# Tribe Artonini Tarmann, 1994 in Korea (Lepidoptera, Zygaenidae)

**DOI:** 10.3897/zookeys.1285.189091

**Published:** 2026-07-22

**Authors:** Ulziijargal Bayarsaikhan, Konstantin A. Efetov, Gerhard M. Tarmann, Tak-Gi Lee, Jae-Ho Ko, Hyung Wook Kwon

**Affiliations:** 1 Division of Life Sciences, College of Life Sciences and Bioengineering, Incheon National University, Songdo-dong, Incheon 22014, Republic of Korea Sammlungs- und Forschungszentrum der Tiroler Landesmuseen Ferdinandeum Austria; 2 Convergence Research Center for Insect Vectors, Division of Life Sciences, College of Life Sciences and Bioengineering, Incheon National University, Songdo-dong, Incheon 22014, Republic of Korea Convergence Research Center for Insect Vectors, Division of Life Sciences, College of Life Sciences and Bioengineering, Incheon National University Incheon Republic of Korea; 3 Department of Biochemistry and Laboratory of Biotechnology, V. I. Vernadsky Crimean Federal University, Prospekt Vernadskogo 4, Simferopol 29007, Republic of Crimea Division of Life Sciences, College of Life Sciences and Bioengineering, Incheon National University Incheon Republic of Korea; 4 Sammlungs- und Forschungszentrum der Tiroler Landesmuseen, Ferdinandeum, 6060 Hall in Tirol, Austria Research Institute of Basic Sciences, Incheon National University Incheon Republic of Korea; 5 Research Institute of Basic Sciences, Incheon National University, Songdo-dong, Incheon 22012, Republic of Korea Department of Biochemistry and Laboratory of Biotechnology, V. I. Vernadsky Crimean Federal University Simferopol Republic of Crimea; 6 DASARI Research Institute of BioResources, Dunsan-daero, 117 beon-gil, Seo-gu, Daejeon 35203, Republic of Korea DASARI Research Institute of BioResources Daejeon Republic of Korea

**Keywords:** Checklist, distribution, new record, new species, Palaearctic Region, Procridinae

## Abstract

Five species in two genera of the tribe Artonini Tarmann, 1994 (Lepidoptera, Zygaenidae, Procridinae), are recorded from Korea. A new species of the genus *Amuria*, viz. *Amuria baei* Bayarsaikhan & Efetov, **sp. nov**., is described. Illustrations of the adults and genitalia of recorded Korean species of the tribe Artonini are provided, with a checklist of all known Korean zygaenid species, following the classification of Efetov and Tarmann (2024).

## Introduction

The family Zygaenidae is the second largest among the 12 families of the superfamily Zygaenoidea, comprising approximately 1,143 species in 184 genera with a worldwide distribution (Mirić et al. 2024). It consists of five subfamilies, viz: Inouelinae Efetov & Tarmann, 2017; Zygaeninae Latreille, 1809; Callizygaeninae Alberti, 1954; Chalcosiinae Walker, 1865; and Procridinae Boisduval, 1828 (Efetov et al. 2014, 2019a, 2022; Efetov and Tarmann 2017; Mirić et al. 2024).

Commonly known as forester smoky moths, the Procridinae (Efetov 2004, 2005; Yen et al. 2005; Koren 2021) are mainly diurnal and the only subfamily of Zygaenidae with a worldwide distribution (Tarmann 1994; Efetov and Tarmann 2024). According to Tarmann (1994), the subfamily Procridinae can be recognized by the following characters: proboscis developed (reduced in only two genera, *Theresimima* Strand and *Rhagades* Wallengren), chaetosemata present; CuP, analis vein present; ovipositor absent, ductus seminalis connecting to corpus bursae near the orifice, and ductus bursae sometimes with specialized structure (praebursa) in the female genitalia; a pointed uncus without sensory hairs, and well developed valvae in the male genitalia. However, a critical review of these characters shows that none of them can be considered true autapomorphies of the subfamily, as Procridinae share most of them with at least one other subfamily of Zygaenidae (Tarmann 1994, 2004; Efetov and Tarmann 2017). Recently, Efetov and Tarmann (2024) catalogued Procridinae and divided this subfamily into five tribes (Thyrassiini Efetov & Tarmann, 2024; Pollanisini Efetov & Tarmann, 2024; Artonini Tarmann, 1994; Cleleini Efetov & Tarmann, 2024; Procridini Boisduval, 1828) including 570 species in 94 genera in the world. Among them, 171 species were listed from the Palaearctic (including the whole territories of China and Japan) by Efetov and Tarmann (2012).

The first article on the Korean fauna of Zygaenidae was published by Fixsen (1887: 325), who reported one species, *Northia tenuis* Butler, 1877. Since then, subsequent taxonomic studies have gradually clarified the Korean fauna of this family, and most recently *Jordanita mollis* (Grum-Grshimailo, 1893) was recorded by Efetov et al. (2019b), and *Inope heterogyna* Staudinger, 1887 was added by Kim and Choi (2023). Furthermore, one additional species, *Clelea cyanescens* Alberti, 1954, is currently treated as a new record for the Korean fauna by Bayarsaikhan et al. (in press) in a manuscript under review at the "Journal of Asia-Pacific Biodiversity". As a result, Zygaenidae of Korea are currently known to comprise 30 species in 15 genera belonging to three subfamilies.

In the present study, we deal with the genus *Amuria* Staudinger, 1887, first recorded in South Korea, with one new species, *Amuria baei* sp. nov. Additionally, we provide illustrations of the adults and genitalia of the known species of the tribe Artonini from Korea.

## Checklist of known species of the family Zygaenidae from Korea

**Family Zygaenidae**

**Subfamily Procridinae Boisduval, 1828**

**Tribe Artonini Tarmann, 1994**

**Genus *Artona* Walker, 1854**

**Subgenus *Balataea* Walker, 1865**

1. A. (B.) octomaculata (Bremer, 1861)
2. A. (B.) gracilis (Walker, 1865)

**Subgenus *Fuscartona* Efetov & Tarmann, 2012**

1. A. (F.) funeralis (Butler, 1879)
2. A. (F.) martini Efetov, 1997

**Genus *Amuria* Staudinger, 1887**

1. *A. baei* Bayarsaikhan & Efetov, sp. nov.

**Tribe Cleleini Efetov & Tarmann, 2024**

**Genus *Clelea* Walker, 1854**

1. *C. cyanescens* Alberti, 1954

**Genus *Inope* Staudinger, 1887**

1. *I. maerens* (Staudinger, 1887)
2. *I. heterogyna* Staudinger, 1887

**Tribe Procridini Boisduval, 1828**

**Genus *Pseudoilliberis* Efetov & Tarmann, 2012**

1. *P. kuprijanovi* (Efetov, 1995)

**Genus *Illiberis* Walker, 1854**

**Subgenus *Primilliberis* Alberti, 1954**

1. I. (P.) rotundata Jordan, 1907
2. I. (P.) pruni Dyar, 1905

**Subgenus *Illiberis* Walker, 1854**

1. I. (I.) sinensis Walker, 1854
2. I. (I.) assimilis Jordan, 1907

**Subgenus *Euphacusa* Matsumura, 1927**

1. I. (E.) dirce (Leech, 1889)
2. I. (E.) cybele (Leech, 1889)

**Genus *Hedina* Alberti, 1954**

1. *H. nigra* (Leech, 1889)
2. *H. psychina* (Oberthür, 1880)
3. *H. consimilis* (Leech, 1898)
4. *H. hyalina* (Staudinger, 1887)
5. *H. tenuis* (Butler, 1877)

**Genus *Rhagades* Wallengren, 1863**

**Subgenus *Rhagades* Wallengren, 1863**

1. R. (R.) pruni ([Denis & Schiffermüller], 1775)

**Genus *Jordanita* Verity, 1946**

**Subgenus *Roccia* Alberti, 1954**

1. J. (R.) mollis (Grum-Grshimailo, 1893) [see Efetov et al. 2019b]

**Subfamily Chalcosiinae Walker, 1865**

**Tribe Agalopini Alberti, 1954**

**Genus *Elcysma* Butler, 1881**

1. *E. westwoodii* (Snellen von Vollenhoven, 1863)

**Genus *Neochalcosia* Yen & Yang, 1997**

1. *N. remota* (Walker, 1854)

**Tribe Chalcosiini Alberti, 1954**

**Genus *Eterusia* Hope, 1841**

1. *E. aedea sugitanii* Matsumura, 1927
2. *E. watanabei* Inoue, 1982
3. *E. taiwana* (Wileman, 1911)

**Genus *Pidorus* Walker, 1854**

1. *P. atratus* (Butler, 1877)

**Genus *Pseudopidorus* Yen & Yang, 1997**

1. *P. fasciatus* (Felder & Felder, 1862)

**Subfamily Zygaeninae Latreille, 1809**

**Tribe Zygaenini Alberti, 1954**

**Genus *Zygaena* Fabricius, 1775**

**Subgenus *Agrumenia* Hübner, 1819**

1. Z. (A.) niphona Butler, 1877

## Materials and methods

The specimens examined in this study are mainly deposited in the collection of the Incheon National University, Incheon (**INU**), Republic of Korea. One male of Artona (Balataea) gracilis (Walker), adult and genitalia slide no. INU-12997, was collected by one of the corresponding authors (J-HK), through the project of the National Long-Term Ecological Research Program, National Institute of Ecology (**NIE**), Republic of Korea (NIE-B-2025-02). One male of Artona (Balataea) octomaculata (Bremer), adult and genitalia slide no. INU-13040, was collected through Dr Y.-K.Park’s project of the Honam National Institute of Biological Resources (**HNIBR**), funded by the Ministry of Environment (MOE), Republic of Korea (HNIBR202101101). Two specimens of Artona (Fuscartona) martini Efetov, adult and genitalia slide no. INU-13041, 13042, were collected through the project of the authors UB and J-HK under the Honam National Institute of Biological Resources (HNIBR), funded by the Ministry of Environment (MOE), Republic of Korea (HNIBR202101101). Figures of Artona (Fuscartona) funeralis (Butler), based on Japanese material, were kindly provided by Dr Y Nasu and Mr M Sakurai.

Images of the adults and genitalia were taken with a Nikon D700 digital camera attached to a Nikon AF-S VR Micro-Nikkor 105 mm f/2.8G IF-ED lens (Nikon Co., Japan) and a Leica DM2500 microscope equipped with the Leica ICC50 E (Leica Microsystems GmbH, Wetzlar, Germany). The genitalia were dissected and examined under the Leica EZ4 stereo-microscope (Leica microsystems GmbH, Wetzlar, Germany) and the Nikon SMZ10 stereo-microscope. All examined specimens are deposited in the collection of the Incheon National University (**INU**), Incheon, Republic of Korea. Abbreviations used are as follows: **TS** = type species; **TL** = type locality.

## Taxonomic account

### Family Zygaenidae

Taxon classificationAnimaliaLepidopteraZygaenidae

Latreille, 1809

D2EA6607-3725-5B27-9146-912D94815E4A

#### Type genus.

*Zygaena* Fabricius, 1775.

### Subfamily Procridinae

Taxon classificationAnimaliaRosalesZygaenidae

Boisduval, 1828

122CD59C-0C08-58F0-9B83-4B5629C0BACF

#### Type genus.

*Procris* Fabricius, 1807.

### Tribe Artonini

Taxon classificationAnimaliaLepidopteraZygaenidae

Tarmann, 1994

B074F425-F64D-5E16-B151-BC1174DB0199

#### Type genus.

*Artona* Walker, 1854.

#### Notes.

The tribe Artonini was established by Tarmann (1994) with *Artona* Walker, 1854, as the type genus. It comprises approximately 86 species, 12 genera, and five subgenera (Efetov and Tarmann 2024). The members of this tribe are primarily restricted to the Afrotropical and Indo-Australian regions, although a few species also occur in the eastern Palaearctic region (Tarmann 2004).

The latest Korean insect checklist by KSAE and ESK (2021) includes three species of two genera of this tribe, viz. *Balataea* Walker, 1865, and *Fuscartona* Efetov & Tarmann, 2012. Byun et al. (2010) reported one genus of Artonini, viz. *Artona* Walker, 1854, with a single species, *Artona martini* Efetov, 1997, from Seoul, Korea. However, this species was omitted in the KSAE and ESK (2021) checklist. According to Efetov and Tarmann (2024), the two previously recorded genera in Korea are now treated as subgenera within *Artona* Walker. In this study, we report an additional genus, *Amuria* Staudinger, 1887, with a new species, *A. baei* sp. nov. from Goeje Island, Korea. Totally five species of two genera from the tribe Artonini are currently known from Korea.

#### Diagnosis.

Head dorsoventrally compressed, chaetosema extended between compound eye and ocellus; forelegs have epiphysis and single medial spur in hindlegs; in male genitalia, valva has the ‘*Artona*-finger’; in female genitalia, sclerotized praebursa has a double row of pointed spines (Efetov and Tarmann 2024).

#### Host plants.

Poaceae, Zingiberaceae, Pittosporaceae, Lauraceae, Musaceae, Arecaceae.

### Genus Artona

Taxon classificationAnimaliaLepidopteraZygaenidae

Walker, 1854

36B0D02E-E144-5848-88AA-4CFF77353697

TS: Artona discivilla Walker, 1854. TL: North India.

#### Note.

The genus *Artona* Walker was divided into five subgenera by Efetov and Tarmann (2024), viz. *Artona* Walker, 1854, *Zeuxippa* Herrich-Schäffer, 1855, *Balataea* Walker, 1865, *Pseudosesidia* Alberti, 1954, and *Fuscartona* Efetov & Tarmann, 2012. The genus includes 34 described species.

#### Distribution.

According to Efetov and Tarmann (2024: 421–424), all species of the genus *Artona* are distributed across the Oriental and Australasian regions, with occurrences in South, Southeast, and East Asia (including India, Sri Lanka, southern China, Myanmar, Malaysia, Singapore, Indonesia, and the Philippines), as well as Australia (including Queensland and Lizard Island). Some species extend into the eastern Palaearctic and the Pacific islands.

### Artona (Balataea) octomaculata

Taxon classificationAnimaliaLepidopteraZygaenidae

(Bremer, 1861)

E51E809F-034A-524A-9FA8-9EF7EFC2D7CB

**Figures 1–4.**
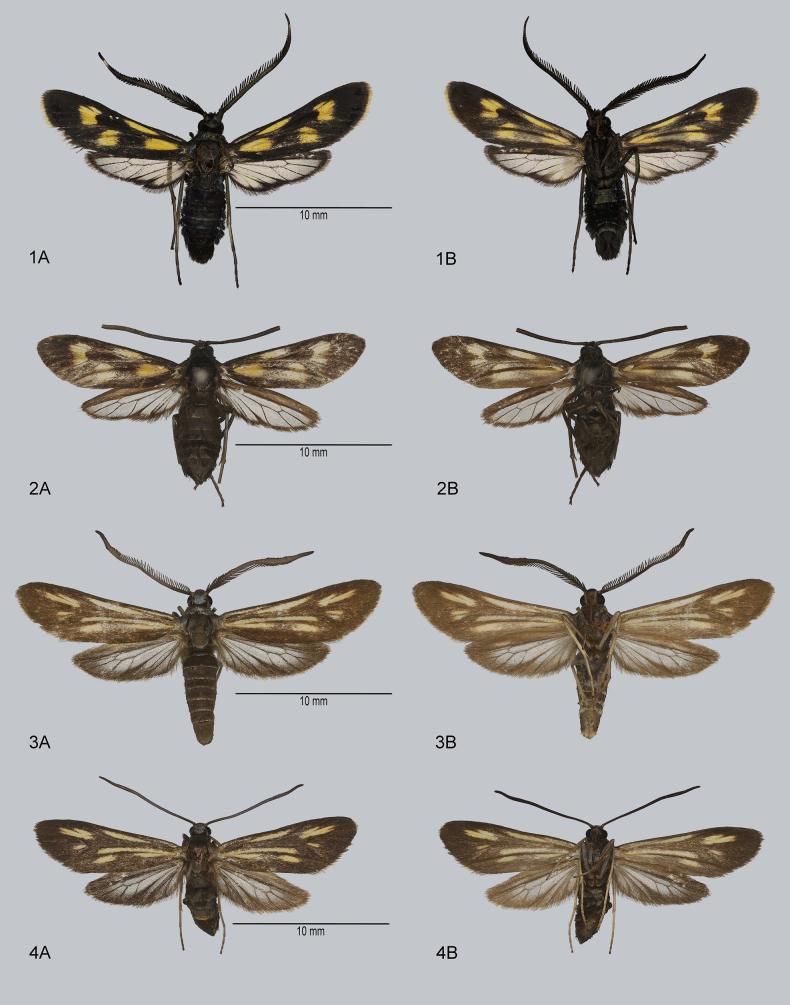
Adults of Korean zygeanid moths. **1A**. Artona (Balataea) octomaculata (Bremer), male, dorsal view (INU-13040K); **1B**. Ditto, male, ventral view (INU-13040K); **2A**. Ditto, female, dorsal view (INU-12995K); **2B**. Ditto, female, ventral view (INU-12995K); **3A**. A. (B.) gracilis (Walker), male, dorsal view (INU-12993K); **3B**. Ditto, male, ventral view (INU-12993K); **4A**. Ditto, female, dorsal view (INU-12992K); **4B**. Ditto, female, ventral view (INU-12992K).

Figs 1A, 1B, 2A, 2B, 9A–C, 15A, 15B

Euchromia octomaculata Bremer, 1861: 476. TL: Russia.

#### Material examined.

1 female, Korea, Gyeonggi-do, Suweon-shi, Mt. Suri, 4 Jul. 2000, Lee, Lee & Yu leg., adult and genitalia slide no. INU-12995K • 1 male, Korea, Jeollanam-do, Wando-gun, *Geumdang-myeon*, 34°26'32"N, 127°03'53"E, *19 Jun. 2025, YK Park leg*., adult and genitalia slide no. INU-13040.

**Figures 5–8.**
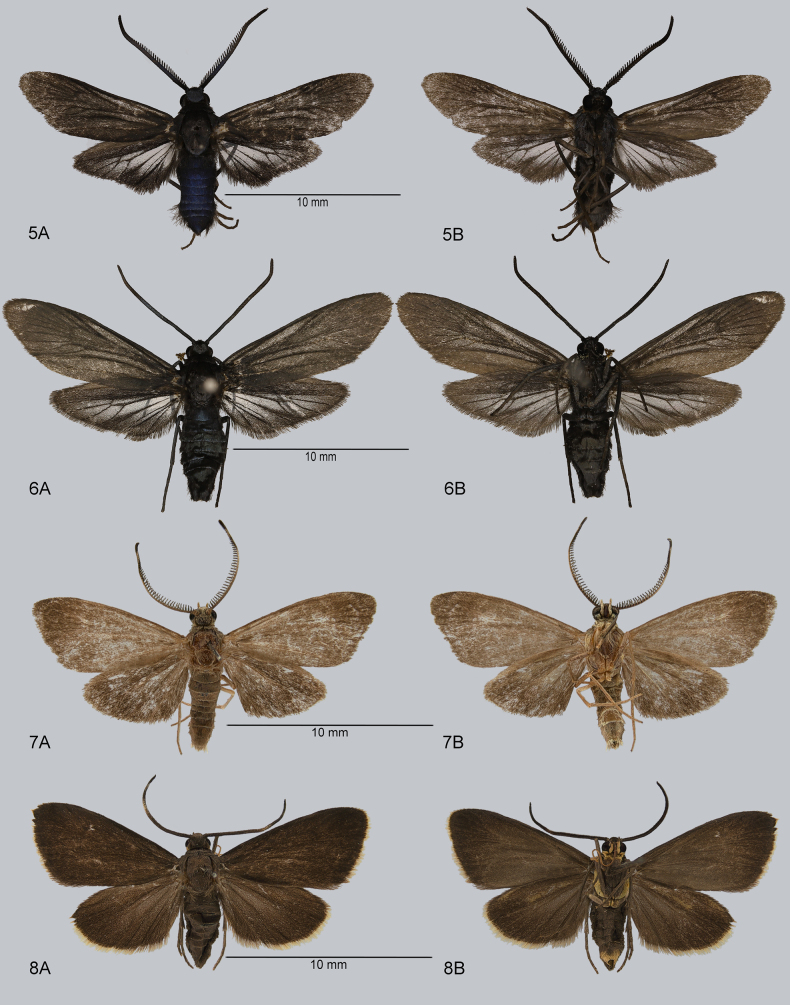
Adults of Korean zygeanid moths. **5A**. Artona (Fuscartona) martini Efetov, 1997, male, dorsal view (INU-13062K); **5B**. Ditto, male, ventral view (INU-13062K); **6A**. Ditto, female, dorsal view (INU-13041K); **6B**. Ditto, female, ventral view (INU-13041K); **7A**. *Amuria baei* sp. nov., holotype, male, dorsal view (INU-12884K); **7B**. Ditto, holotype, male, ventral view (INU-12884K); **8A**. Ditto, paratype, female, dorsal view (INU-12885K); **8B**. Ditto, paratype, female, ventral view (INU-12885K).

**Figures 9, 10.**
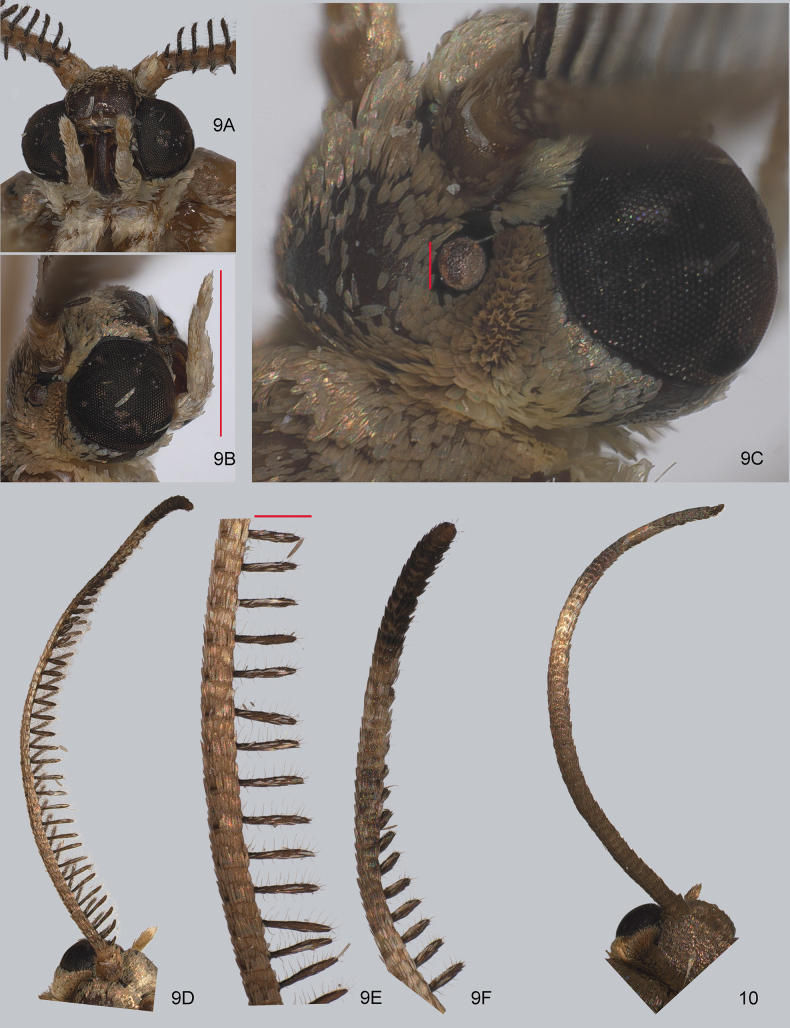
Head of *Amuria baei* sp. nov., holotype, INU-12884K. **9A**. Ventral view, showing a pair of labial palps and coiled proboscis; **9B**. Lateral view, showing upcurved labial palps and compound eye; **9C**. Lateral view, showing ocellus and chaetosema; **9D**. Male antenna; **9E**. Pectinations in middle part of antenna in holotype; **9F**. Apical part of antenna in holotype; **10**. Female antenna in paratype, INU-12885K. Scale bars: 1 mm (**B**); 0.1 mm (**C**); 0.5 mm (**E**).

**Figures 11, 12.**
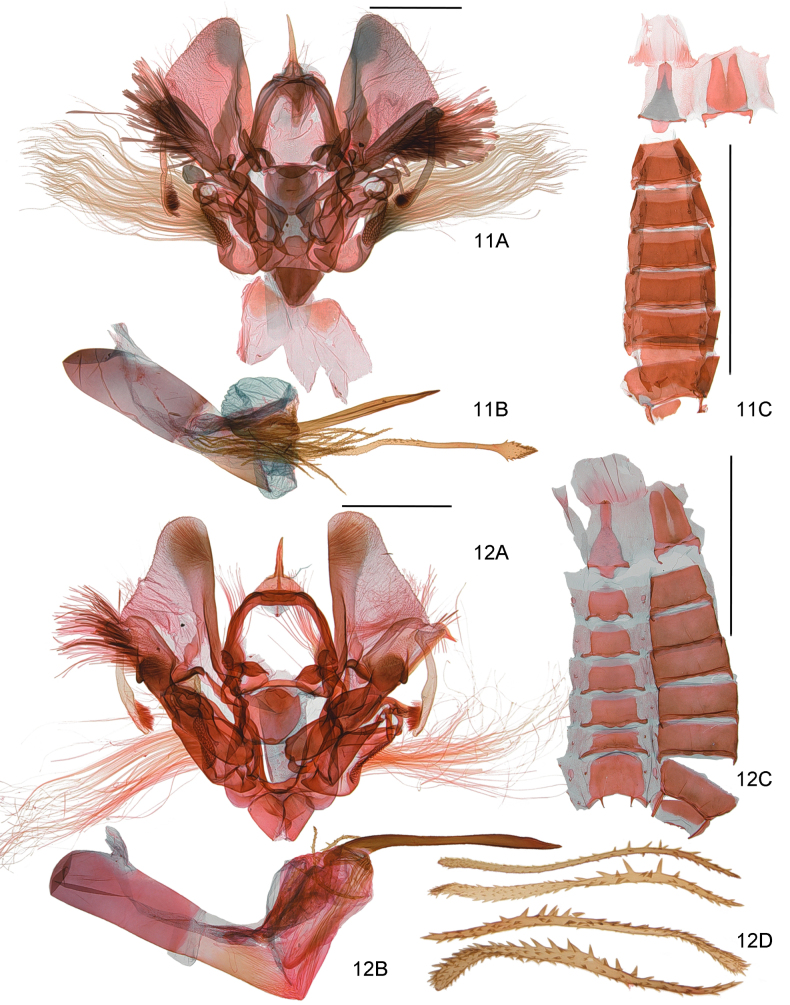
Male genitalia of Korean zygeanid moths. **11A–C**. Artona (Balataea) octomaculata, INU-13040K; **12A–D**. A. (B.) gracilis, INU-12993K. **A**. Genitalia capsule; **B**. Phallus; **C**. Abdomen; **D**. Diverse of cornutus in the vesica. Scale bars: 1 mm (**A, B**); 5 mm (**C**).

**Figures 13, 14.**
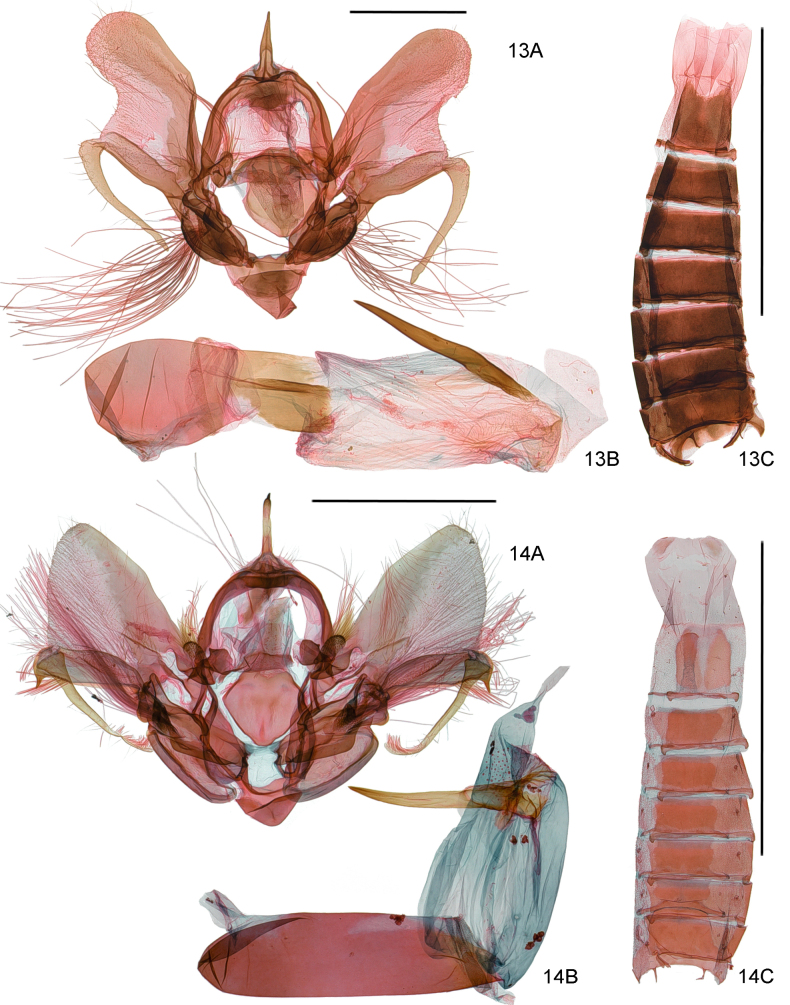
Male genitalia of Korean zygeanid moths. **13A–C**. Artona (Fuscartona) martini, INU-13050K; **14A–C**. *Amuria baei* sp. nov., holotype, INU-12884K. **A**. Genitalia capsule; **B**. Phallus; **C**. Abdomen. Scale bars: 1 mm (**A, B**); 5 mm (**C**).

**Figures 15–18.**
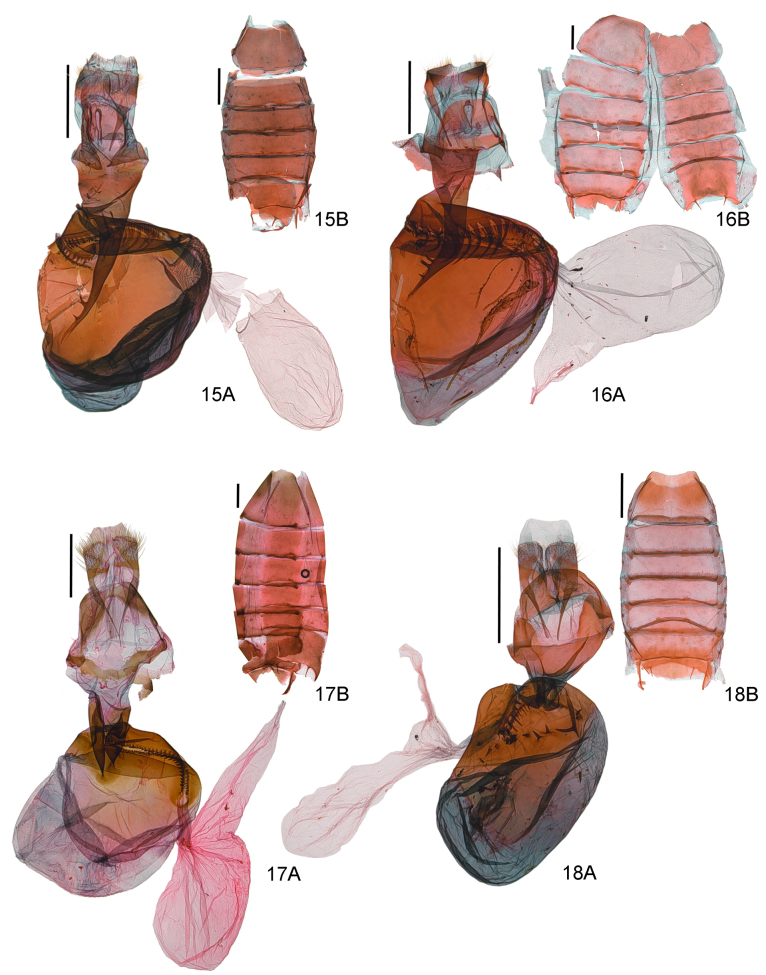
Female genitalia of Korean zygeanid moths. **15A–C**. Artona (Balataea) octomaculata, INU-12995K; **16A–C**. A. (B.) gracilis, INU-12994K; **17A–C**. (Fuscartona) martini, INU-13042K; **18A–C**. *Amuria baei* sp. nov., paratype, INU-13065K. Scale bar: 1 mm.

#### Diagnosis.

Wingspan 9–11 mm in males, 11–12 mm in females. This species differs from its congeners by the antennae being dark brown with a distinct yellowish white band near the apex; the head, thorax, and abdomen dark brown, slightly covered with bluish scales; the forewing with a dark brown ground color bearing stout, short, pure yellow patches, and the cilia pure yellow, whereas the hindwing is brownish with the central area semitransparent, and the cilia brownish; legs dark brown, slightly covered with bluish scales. Male genitalia with valva broadly triangular, the costal margin weakly sclerotized and sparsely setose; juxta weakly sclerotized and distinctly trapezoid. Female genitalia with proximal part of ductus bursae broad, sclerotized; remainder of ductus bursae strongly dilated, forming strongly sclerotized, irregularly globose praebursa, bearing a long longitudinal double row of long tooth-like sclerotizations; ductus intrabursalis short, narrow, translucent; corpus bursae membranous, ovoid with folded walls.

#### Distribution.

Korea, Far East of Russia, China, Japan (Leech 1888; Efetov 2005; Owada and Inada 2005; Hirowatari et al. 2013; KSAE and ESK 2021; Efetov and Tarmann 2024).

#### Host plants.

Efetov (2016) noted *Panicum crusgalli* (Poaceae) and *Molinia japonica* (Cyperaceae). In Korea, Heo (2012: 167) and Kim and Kim (2022: 167) provided illustrations of the larva, pupa, and adult of ‘*Balataea*’ *octomaculata* and reported Poaceae (*Miscanthus* sp. and *Phragmites* sp.) as the host plants of this species.

#### Remarks.

This species was first recorded from Gensan [Wonsan], North Korea by Leech (1888: 594) as *Balataea octomaculata* (Bremer, 1861). Subsequently, Okamoto (1924: 145) reported it from Suwon, South Korea. According to Efetov and Tarmann (2024: 423), the species is now placed in the subgenus *Balataea* Walker, 1865, within the genus *Artona* Walker, 1854.

### Artona (Balataea) gracilis

Taxon classificationAnimaliaLepidopteraZygaenidae

(Walker, 1865)

F5E8E13B-1B5F-56B4-8360-CBF173821BDD

Figs 3A, 3B, 4A, 4B, 12A–D, 16A, 16B

Bintha gracilis Walker, 1865: 127. TL: Japan.

#### Material examined.

1 male, 2 females, Korea, Gangwon-do, Pyengchang-gun, Mt. Odae, 8 Jul. 1998, Bae, Ahn, and Kim leg., adult and genitalia slide no. INU-12992 (female), 12993 (male), 12994 (female) • 2 males; Gangwon-do, Inje-gun, Girin-myeon, Gombaeryeong-gil 20; 38°02'16.9"N, 128°28'04.9"E; 2 Jul. 2024; Ko J.-H. and Lee T.-G. leg.; adults and genitalia slides no. INU-12996, 12997.

#### Diagnosis.

Wingspan 11–12 mm in males, 10–13 mm in females. This species differs from its congeners by the antennae being uniformly brown, lacking a pale band; the head, thorax, and abdomen brownish, slightly covered with bluish scales, and each abdominal segment bearing a thin pale band; the forewing with a brownish ground color bearing slender pale yellow patches, and the cilia pale brownish yellow, whereas the hindwing is brownish with the central area semitransparent, and cilia brownish; legs pale brownish yellow. Male genitalia with valva narrowly triangular, with a broadly rounded apex, costal margin broadly sclerotized and sparsely setose; juxta weakly sclerotized and rounded. Female genitalia with proximal part of ductus bursae narrow, short, weakly sclerotized; remainder of ductus bursae strongly dilated, forming strongly sclerotized, ovoid praebursa, bearing a long longitudinal double row of long tooth-like sclerotizations; ductus intrabursalis short, narrow, translucent; corpus bursae membranous, ovoid with folded walls.

#### Distribution.

Korea, Far East of Russia, eastern China, Japan (Leech 1888; Efetov 2005; Owada and Inada 2005; Hirowatari et al. 2013; KSAE and ESK 2021; Efetov and Tarmann 2024).

#### Host plants.

Efetov (2016) noted Poaceae as the host plant of this species. In Korea, no data are available on the host plants of this species.

#### Remarks.

This species was first recorded from Gensan [Wonsan], North Korea by Leech (1888: 594) as *Bintha gracilis* Walker, 1865. Shin et al. (1983) recorded this species based on a specimen collected by Kim in 1961 from Mt. Jiri (Nogodan), South Korea, which is confirmed to be the first documented locality for this species in South Korea. Recently, the species was placed into the subgenus *Balataea* Walker, 1865, within the genus *Artona* Walker, 1854, by Efetov and Tarmann (2024: 423).

### Artona (Fuscartona) martini

Taxon classificationAnimaliaLepidopteraZygaenidae

Efetov, 1997

5AC840FE-2ECA-5DAF-8D01-EBA483D92BA8

Figs 5A, 5B, 6A, 6B, 13A–C, 17A, 17B

Artona (Fuscartona) martini Efetov, 1997: 170. TL: Japan.

#### Material examined.

3 females, Korea, Jeollanam-do, Sinan-gun, Anjwa-myeon, Anjwado Island, 6 Jun. 2025, Bayarsaikhan U. & Ko J.-H. leg., adults and genitalia slides no. INU-13041, 13042, 13043. 1 male, Korea, Chungcheongnam-do, Dangjin-si, Dangjin 1(il)-dong, San 47, 6 Jul. 2019, Paek Munki leg./Det., adult no. INU-13051 • 1 female, Korea, Incheon, Yeonsu-gu, Neungheo-daero 437, 23 Jul. 2025, Bayarsaikhan U. and Lee T.-G. leg., adult and genitalia slide no. 13043 • 2 males, 5 females, Korea, Incheon, Jung-gu, Wolmi-ro 131-22, 23 Jul. 2025, Ko J.-H leg • 2 males, 2 females, Incheon, Namdong-gu, Jeonggak-ro 9, 8 Sep. 2025, Lee T.-G. leg., adults and genitalia slides no. INU-13049, 13050, 13062, 13063.

#### Diagnosis.

Wingspan 16–18 mm in males, 21–23 mm in females. This species differs from its congeners by the antennae, head, thorax, and abdomen being dark brown to blackish and distinctly covered with bluish metallic scales; the forewing deep black, tinged with dark grey, and the hindwing rather paler, with the central area semitransparent; cilia dark brown on both wings; legs dark brown to blackish. Male genitalia with valva narrowly reniform without pointed process at apex, costal margin broadly sclerotized and sparsely setose; *Artona*-finger (movable process near the apex of sacculus, see Efetov and Tarmann 2024) pointed at apex, without apical brush of setae at the apex; phallus stout, with the vesica bearing a large spine-like cornutus, nearly equal in length to the phallus. Female genitalia with proximal part of ductus bursae narrow, laterally strongly sclerotized; remainder of ductus bursae strongly dilated, forming strongly sclerotized ovoid praebursa, bearing a long longitudinal double row of short tooth-like sclerotizations; ductus intrabursalis short, narrow, translucent; corpus bursae small, membranous, ovoid with folded walls.

#### Distribution.

Korea, China, Taiwan, Vietnam, Japan, New Zealand, Italy (Efetov 1997; Owada and Inada 2005; Byun et al. 2010; Hirowatari et al. 2013; Marianelli et al. 2020; KSAE and ESK 2021; Efetov and Tarmann 2024).

#### Host plants.

According to Efetov (1997) and Gill (2000), several bamboo species, *Bambusa multiplex*, *Shibataea kumasasa*, *Pleioblastus viridistriatus*, *Phyllostachys nigra*, and *Miscanthus sinensis*, were noted as host plants of this species in China and New Zealand. In Korea, Byun et al. (2010) reported Poaceae (bamboo) as the host plant.

#### Remarks.

This species was first recorded from Seoul, Korea by Byun et al. (2010) as *Artona martini* Efetov, based on a study of the immature stages and host plant. However, the Korean record was omitted from subsequent Korean insect checklists by Paek et al. (2010) and KSAE and ESK (2021), as well as from the global checklist compiled by Efetov and Tarmann (2024). The immature stages of this species were later restudied in Korea by Heo (2012: 164) as *Artona martini* Efetov, without assigning a Korean common name. Subsequently, Kim and Kim (2022: 168) also investigated the immature stages under the name *Fuscartona martini* (Efetov) and proposed the Korean name “Dae-na-mu-sswaegi-allaknabang”. However, this Korean name has historically been applied to *Artona funeralis* (Butler) in all Korean insect checklists published between 1968 and 2024.

### Genus Amuria

Taxon classificationAnimaliaLepidopteraZygaenidae

Staudinger, 1887

AF7B806E-DD29-538B-8C72-FB38DC2E42C9

TS: Amuria cyclops Staudinger, 1887. TL: Russia.

#### Note.

The genus *Amuria* Staudinger, 1887 belongs to the tribe Artonini and currently comprises 21 species (Efetov and Tarmann 2024). The type species, *Amuria cyclops* Staudinger, 1887, was originally described from the Amur region, with the type locality explicitly given as Vladivostok by Staudinger (1887: 172). Leech (1888: 597) subsequently mentioned this species in a brief note, stating that he had not examined any specimens himself and merely followed the original description; he listed the distribution as Vladivostok, Askold, Sidemi, and Corea [Korea]. Kirby (1892: 89) later restricted the distribution to Vladivostok only. In contrast, Leech (1898: 332) examined a single male specimen from Chang-yang, Central China, and accordingly gave the distribution as Amurland and Central China, omitting Korea (Corea). Nevertheless, several later authors, Staudinger and Rebel (1901: 391), Jordan (1907: 14), Bryk (1936: 248), and Efetov (2016: 219), continued to include Korea (Corea) in the distribution of *A. cyclops*, apparently without examination of any Korean voucher specimens. Among these works, Alberti (1954: 273) stands out in that he examined Staudinger’s type material and provided illustrations of the adult and genitalia of *A. cyclops*.

To date, however, no confirmed specimen of the genus *Amuria* has been verified from Korea. Therefore, based on the discovery and description of *Amuria baei* sp. nov. in the present study, the genus *Amuria* is recorded from Korea for the first time.

#### Diagnosis.

The habitus is dominated by dark (mainly brown, purplish, or bluish) colors. Some species are almost uniformly dark and if there is a pattern the dark colors contrast with white or light yellow markings. The wings are broad (vs narrow in *Artona*). The typical yellow and black wing pattern of the subgenera *Artona* and *Zeuxippa* of the genus *Artona* is absent and the body is never ringed with yellow color like in most Artona (Artona) and Artona (Zeuxippa) species. In the male genitalia the *Artona*-finger is present, the apex of the valva bears spiny prolongations (found also in Artona (Balataea), but absent in the subgenus *Artona*). The female genitalia of all *Amuria* species have well developed praebursa with a longitudinal double row of short tooth-like sclerotizations (Efetov and Tarmann 2024).

#### Distribution.

According to Efetov and Tarmann (2024: 425–427), species of the genus *Amuria* are distributed in Southeast and East Asia: Russian Far East (Khabarovsk and Primorsky Krai), Korea, northeastern China, northeastern India, Myanmar, Indonesia, New Guinea with adjacent territories and Australia (Queensland).

#### Host plants.

Nazari et al. (2019) and Efetov and Tarmann (2024) mentioned Zingiberaceae, Musaceae, Pittosporaceae, and Lauraceae as the host plants of the genus *Amuria* Staudinger.

### Amuria baei

Taxon classificationAnimaliaLepidopteraZygaenidae

Bayarsaikhan & Efetov sp. nov.

44DD87D2-0FB0-50D9-A9F8-9DEF22A5D446

https://zoobank.org/8E20FAAC-2192-40B1-B959-4F1A345C45C7

Figs 7A, 7B, 8A, 8B, 9A–C, 10, 14A–C, 18A, 18B

#### Type material.

***Holotype***. 1 male, Korea, Gyeongsangnam-do, Is. Geoje, Mt. Noja, 22–26 Jun. 1998, 34°47'18.6"N, 128°36'56.1"E, Y.-S. Bae et al. leg., adult and genitalia slide no. INU-12884; deposited in INU. ***Paratypes***. 1 male, 5 females, same collection data as the holotype, adults and genitalia slides no. INU-12927male, 12928female, 13065female • 4 females, Korea, Gyeongsangbuk-do, Younju-shi, Mt. Seonda, 29 Jun. 1998, Bae Y.-S. and Paek M.-K. leg., adult and genitalia slide no. INU-12885female, 13064female; deposited in INU.

#### Diagnosis.

By the wing pattern of this species, this new species is hardly distinguishable from other Korean zygaenid moths, particularly those of the genus *Inope* Staudinger, 1887. Both of these genera have a dark brown ground wing color without distinct patterns, as well as similar body coloration and size. Specimens of the new species were previously mixed and misidentified as *Inope maerens* (Staudinger, 1887) in the INU collection. This misidentification is the reason the species has remained undescribed since its collecting in 1998. However, *Amuria baei* sp. nov. differs from *Inope* species by having a more sharply angled forewing apex and a pale yellowish band near the distal end of the antennae in both sexes; the ventral side of thorax weakly covered with yellowish scales, the labial palpus pale yellowish, except pale brown first segment.

There are only two species of *Amuria* known, viz. *Amuria chorista* (Jordan, 1908) and *A. lugubris* (Jordan, 1908), with a similar habitus as *Amuria baei* sp. nov. All three species have uniformly dark wings on the upperside and without or almost no pattern on the underside. *Amuria chorista* is easily distinguished from *A. baei* by its narrower wings, bright yellow proboscis, and the dark fringe of the wings. *Amuria lugubris* is smaller, has as well a dark proboscis and a yellowish fringe like *A. baei* but the wings are even more narrow than those of *A. chorista*, the ground color is not brownish but more greyish black, and *A. lugubris* also has a purple sheen on wings and body. Moreover, the underside of the wings is paler in color than the upperside and a yellowish pattern in the form of a stripe on the distal part of the costa and at cell on the hindwing underside are visible. In *A. lugubris* the whole underside of the body, from the palpi on the head to the end of the abdomen is yellow, whereas in *A. baei* only the palpi, parts of thorax and legs, and the last segment of the abdomen are brownish yellow. *Artona chorista* and *A. lugubris* are only known from northeastern India (so far).

#### Description.

**Male** (Fig. 7A, B). Forewing length 7.5–8 mm (holotype 8 mm), forewing breadth 3–3.5 mm (holotype 3.5 mm); hindwing length 5–6 mm (holotype 6 mm), hindwing breadth 3–3.5 mm (holotype 3 mm); length of body 5.5–6 mm (holotype 6 mm); length of antenna 4.5–5 mm (holotype 5 mm). Female (Fig. 8A, B). Forewing length 7.5–8 mm, forewing breadth 3–4 mm; hindwing length 5.5–6 mm, hindwing breadth 3–3.5 mm; length of antenna 4.5–5.5 mm. Habitus of males and females similar. Frons protruding forward (Fig. 9A). Compound eyes black (Fig. 9A, B). Chaetosema triangular, protruding forwards between compound eye and ocellus, brown (Fig. 9C). Ocellus brown (Fig. 9C). Proboscis brown, well developed (Fig. 9A). Palpi pale brownish yellow, long (Fig. 9A, B). Antenna bipectinate in male, length of pectinations in middle part of antenna in males 0.5 mm (holotype 0.5 mm), antenna with 45 segments in male (16 distal segments biserrate) and biserrate in female (Figs 9D, 9E, 9F, 10). Thorax, forewing, and hindwing upperside and underside as well as abdomen brown. Fringes of wings yellow. Femura of legs, parts of thorax and tip of last segment of abdomen ventrally brownish yellow. Foreleg with tibial epiphysis, midleg with one pair of spurs (apical), hindleg with three spurs (one middle and two apical).

**Male genitalia** (Fig. 14A, B, C). Uncus single, narrow, with pointed apex, sclerotized. Valva triangular, fan-shaped, with rounded apex, slightly sclerotized dorsally and strongly sclerotized ventrally, apex of sacculus with small pointed process and characteristic *Artona*-finger with curved apex (apex of finger covered with a brush of setae). Juxta sclerotized, subquadrate. Phallus long, more than 3 times longer than uncus, straight, vesica with one large sclerotized cornutus with pointed apex.

**Female genitalia** (Fig. 18A, B). Ostium bursae roundish with sclerotization only dorsally; proximal part of ductus bursae short, cone shaped, translucent; remainder of ductus bursae strongly dilated, forming sclerotized ovoid praebursa, bearing a double rows of long tooth-like sclerotizations, there is also additional group of pointed spines on the walls of praebursa; distal part of praebursa translucent; ductus intrabursalis narrow, translucent; corpus bursae membranous, ovoid with folded walls, without signa.

#### Distribution.

South Korea (Is. Geoje, Gyeongsangnam-do and Mt. Seondal, Gyeongsangbuk-do).

#### Host plants.

No data are available on the host plants of this new species.

#### Etymology.

The specific name is dedicated to Prof. Yang-Seop Bae (Incheon National University, Republic of Korea), in gratitude for his mentorship during the graduate studies of several authors of this paper and in recognition of his outstanding contributions to the taxonomy and biodiversity of Lepidoptera in Asian countries.

### Artona (Fuscartona) funeralis

Taxon classificationAnimaliaLepidopteraZygaenidae

(Butler, 1879)

3FBCC8D4-D9F6-5770-B9C8-5021F9DF2D72

**Figures 19, 20.**
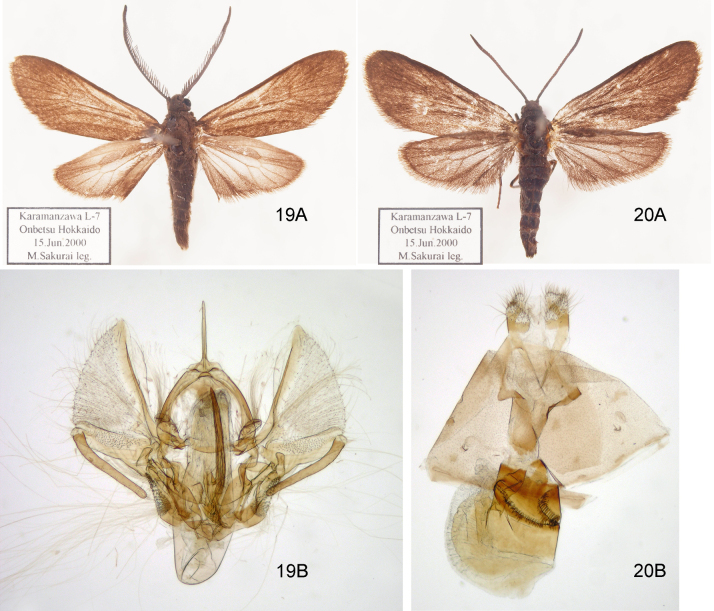
Artona (Fuscartona) funeralis (Butler, 1879) from Japan. **19A**. Male, YN-3832, M. Sakurai leg.; **19B**. Male genitalia, YN-3832, Y. Nasu del.; **20A**. Female, YN-3833, M. Sakurai leg.; **20B**. Female genitalia, YN-3833, Y. Nasu del.

Figs 19A, 19B, 20A, B

Procris funeralis Butler, 1879: 351. TL: Japan.

#### Note.

Following the original description by Butler (1879: 351, in English): deep purplish brown, the fringes paler; the disc of secondaries whitish and semi-hyaline; abdomen black; claspers and proboscis horn-yellow; a whitish extruded anal tuft. Expanse of wings 9 lines. The description of this species was corrected by Efetov (1997: 168) based on the holotype examination. The holotype is a female with the male genitalia (mentioned by Butler as ‘a whitish extruded anal tuft’) on the end of the abdomen connected with the female genitalia of the holotype.

#### Material examined.

Japan • 1 male, 1 female; Karamanzawa L-7, Onbetsu Hokkaido; 15 Jun. 2000; M. Sakurai leg.; adult and genitalia slide no. YN-3832male, YN-3833female.

#### Distribution.

?Korea, Far East of Russia (Sakhalin and Southern Kuril Islands), China, Japan (Efetov 1997, 2016; Hirowatari et al. 2013; KSAE and ESK 2021; Efetov and Tarmann 2024).

#### Host plants.

According to Efetov (2016) and Hirowatari et al. (2013), *Sasa* species (*Sasa kurilensis*) of the family Poaceae, was noted as host plants of this species.

#### Remarks.

This species was first recorded from Gensan [Wonsan], Korea (currently North Korea) by Leech (1898: 331) under the name *Adscita funeralis* Butler, 1879. Subsequently, it was listed as *Artona funeralis* Butler by the Zoological Society of Korea (1968: 55), as *Balataea funeralis* (Butler, 1879) by Shin et al. (1983: 291), ESK and KSAE (1994: 339), and Paek et al. (2010: 277), and as *Fuscartona funeralis* (Butler, 1879) by KSAE and ESK (2021: 523); however, none of these publications provided illustrations of the adult or genitalia. Park et al. (2012) presented the first illustration of the adult, apparently a female, together with a brief description and ecological information, largely consistent with those given by Shin et al. (1983). All of the above-mentioned publications used a Korean common name for this species.

When the original description of this species by Butler (1879), as well as the redescription by Efetov (1997), are compared with the brief descriptions in the Korean literature (Shin et al. 1983; Park et al. 2012), the Korean specimens are described as having a black ground color of the body and wings, with a slight bluish sheen on the body. This discrepancy suggests that species of Artona (Fuscartona) martini Efetov, 1997, may have been misidentified as Artona (Fuscartona) funeralis (Butler, 1879) in the Korean literature. A similar taxonomic confusion was also clarified by Hirowatari et al. (2013: 326) through their examination of Japanese material, in which they demonstrated that the name *funeralis* had long been misapplied to two distinct species and that A. (Fuscartona) martini represents a separate taxon with the type locality in Nagoya, Japan.

Despite examinations of domestic institutional and private collections, as well as additional field surveys, no confirmed Korean specimens of *A. funeralis* (Butler) were found. Therefore, Japanese material was examined in the present study.

## Discussion

The present study provides the first comprehensive treatment of the tribe Artonini in Korea, including the description of a new species and clarification of previous taxonomic ambiguities. As a result, five species belonging to two genera are currently confirmed from the Korean Peninsula. The discovery of *Amuria baei* sp. nov. represents the first confirmed record of the genus *Amuria* from Korea. Although earlier literature occasionally listed Korea within the distribution of *A. cyclops*, no verified voucher specimens have been documented. The present study demonstrates that previous references to Korea were likely based on secondary citations rather than examination of Korean material. Therefore, the occurrence of *Amuria* in Korea is formally established here from specimen-based evidence.

The taxonomic status of Artona (Fuscartona) funeralis in Korea requires reconsideration. Historical Korean records consistently listed this species; however, none of the published accounts provided diagnostic illustrations or genitalia examinations. A comparison of the original description of *A. funeralis* and subsequent redescription with Korean literature suggests that the species previously identified as *funeralis* in Korea corresponds instead to A. (F.) martini. This interpretation is supported by the bluish metallic sheen and genital morphology observed in Korean specimens. Furthermore, no confirmed Korean material of *A. funeralis* was found during extensive examinations of institutional and private collections. The use of Japanese reference material in this study further supports the absence of verified Korean specimens. Consequently, the occurrence of *A. funeralis* in Korea remains doubtful and requires confirmation based on voucher specimens.

The confirmed presence of *A. martini* in Korea is reinstated based on examined material and genitalia comparison. Its omission from some recent Korean checklists appears to have resulted from historical taxonomic confusion rather than true absence. The clarification provided here stabilizes the nomenclature of Korean Artonini and aligns the national fauna with current global taxonomy.

Biogeographically, the confirmed Korean species of Artonini are restricted mainly to the southern part of the peninsula, including Geoje Island. The occurrence of *Amuria* and *Artona* species in these regions indicates that southern coastal areas of Korea play an important role in maintaining the diversity of Artonini. The present data suggest that the Korean fauna represents the northernmost documented distribution of certain taxa within the tribe in East Asia. However, additional faunistic surveys and molecular analyses are required to better understand the origin and internal differentiation of Korean populations.

In conclusion, this study refines the taxonomic framework of Korean Artonini, establishes the first confirmed national record of the genus *Amuria*, describes a new species endemic to Korea, and resolves long-standing misidentifications in the Korean literature. These findings contribute to nomenclatural stability the and provide an updated baseline for future systematic and faunistic studies of Zygaenidae in Korea.

## Supplementary Material

XML Treatment for Zygaenidae

XML Treatment for Procridinae

XML Treatment for Artonini

XML Treatment for Artona

XML Treatment for Artona (Balataea) octomaculata

XML Treatment for Artona (Balataea) gracilis

XML Treatment for Artona (Fuscartona) martini

XML Treatment for Amuria

XML Treatment for Amuria baei

XML Treatment for Artona (Fuscartona) funeralis

## Additional information

### Artificial Intelligence (AI) use

The authors accept full responsibility for the content of the manuscript, including the disclosure of any use of AI.

No AI tools were used in the preparation of this manuscript.

### Author ORCIDs

K.A. Efetov https://orcid.org/0000-0003-1468-7264

T.-G. Lee https://orcid.org/0000-0002-6666-5967

J.-H. Ko https://orcid.org/0000-0001-6557-4281

H.W. Kwon https://orcid.org/0000-0001-9340-7974

### Data availability

All examined specimens are deposited in the collection of Incheon National University (INU), Republic of Korea. All data supporting the findings of this study are included in the article, including specimen data, locality information, and morphological documentation. No additional datasets were generated or analyzed during the current study.
